# Changes in Soybean Global Gene Expression after Application of Lipo-Chitooligosaccharide from *Bradyrhizobium japonicum* under Sub-Optimal Temperature

**DOI:** 10.1371/journal.pone.0031571

**Published:** 2012-02-13

**Authors:** Nan Wang, Wajahatullah Khan, Donald L. Smith

**Affiliations:** 1 Department of Plant Science, McGill University, Ste Anne de Bellevue, Quebec, Canada; 2 Genome Research Chair Unit, Biochemistry Department, College of Science, King Saud University, Riyadh, Saudi Arabia; University of Leeds, United Kingdom

## Abstract

Lipo-chitooligosaccharides (LCOs), signal compounds produced by N_2_-fixing rhizobacteria after isoflavone induction, initiate nodule formation in host legumes. Given LCOs' structural similarity to pathogen-response-eliciting chitin oligomers, foliar application of LCOs was tested for ability to induce stress-related genes under optimal growth conditions. In order to study the effects of LCO foliar spray under stressed conditions, soybean (*Glycine max*) seedlings grown at optimal temperature were transferred to sub-optimal temperature. After a 5-day acclimation period, the first trifoliate leaves were sprayed with 10^−7^ M LCO (NodBj-V (C_18∶1_, MeFuc)) purified from genistein-induced *Bradyrhizobium japonicum* culture, and harvested at 0 and 48 h following treatment. Microarray analysis was performed using Affymetrix GeneChip® Soybean Genome Arrays. Compared to the control at 48 h after LCO treatment, a total of 147 genes were differentially expressed as a result of LCO treatment, including a number of stress-related genes and transcription factors. In addition, during the 48 h time period following foliar spray application, over a thousand genes exhibited differential expression, including hundreds of those specific to the LCO-treated plants. Our results indicated that the dynamic soybean foliar transcriptome was highly responsive to LCO treatment. Quantitative real-time PCR (qPCR) validated the microarray data.

## Introduction

The legume-rhizobia N_2_-fixation symbiosis is one of the most intensively studied and best characterized plant-microbe interactions because it is the most inexpensive and environmental-friendly source of nitrogen for crop production [1]. The establishment of the symbiotic relationship involves a signal exchange between the host legume and the N_2_-fixing rhizobia. Legume roots exude (Iso) flavonoids that act as a chemoattractant to rhizobia and induce the rhizobial *nod* genes [2], [3]. As a result, lipo-chitooligosaccharides (LCOs), also known as Nod factors, are synthesized by rhizobia and excreted as the host-specific rhizobia-to-plant signals [4], [5]. They can be perceived by multiple receptors in host roots and trigger a cascade of signaling events, which are essential for bacterial invasion of the host roots, leading to the formation of N_2_-fixing root nodules [6]. The host plant responses upon exposure to LCOs can be categorized as nodulation-related and non-nodulation-related. The former consists of four events: 1) root hair curling and deformation, 2) electrophysiological responses including ion fluxes, 3) formation of infection threads and development of nodules, 4) activation of early *nodulin* (*enod*) genes in host plants, which encode proteins responsible for early nodule development [5], [7]–[10]. LCOs are viewed as a potential class of plant growth regulators [5], and are known to participate in plant development and morphogenesis [11]–[19], as well as abiotic and biotic stress responses [20]–[22]. Hence, the effects of LCOs outside the context of nodulation are of particular interest.

Microarray studies have previously been carried out on several legume-rhizobia symbiotic systems, illustrating differentially expressed plant genes due to rhizobia infection, nodule development and the onset of N_2_-fixation [23]–[25]. The numerous differentially expressed genes identified in various studies are diverse in class, function, expression level and pattern, however, in general 1) nodulation strongly affects metabolism, 2) plant defense mechanisms are engaged, particularly during the early stages of rhizobial infection, 3) genes usually involved in a variety of regulatory components, such as transcription factors, are affected [24], [26]–[28]. Nonetheless, the profile of the host transcriptome upon the perception of LCOs in the absence of rhizobia has not been well characterized.

Lindsay [12] found that under optimal growth conditions over 600 soybean genes were differentially expressed 48 h after foliar spray with of LCO (NodBj-V (C_18∶1_, MeFuc), the bulk of these being related to defense and stress responses. *Nodulin* homologues were also found to be differentially expressed, indicating the possible presence of LCO receptors in the leaves [12]. In contrast to root application, foliar application of LCOs is a relatively new approach for studying their effects and possible roles. It is quite intriguing that a signal compound from the rhizosphere can trigger responses when applied elsewhere on a plant. However, this novel finding facilitates investigation of LCO effects outside the context of nodulation. Among the thousands of differentially expressed genes revealed so far by microarray investigations of nodulating roots, a significant percentage are clearly nodulation-related [24]. Because foliar application of LCOs will not lead to the formation of nodules, it will allow us to elucidate general LCO responses, in the absence of specific nodulation related responses. Furthermore, the early signaling events in roots following the exposure of LCOs are highly localized, with only a small portion of the root tissue responding. In this regard, harvesting the entire root system may result in tissue dilution, while separating the root sections at harvest are not practically achievable [24]. Using leaf material avoids such problems and may increase the sensitivity of gene expression detection. Moreover, leaves are above-ground and clearly visible, hence it is easier to control the uniformity of the material and the amount of LCO spray applied, which are important in reducing variability in the microarray data analysis.

A number of lines of evidence indicate a relationship between LCOs and host stress response in the absence of external stress; hence the present study was conducted to investigate the effect of LCOs under stress. Low temperature is an appropriate stress condition and a common stress factor during early spring growing conditions for soybean in Eastern Canada. It has previously been reported that the symbiotic interaction is temperature-sensitive, especially during rhizobial infection and nodule development [29], yet the rhizobia-legume symbiosis is almost always established under sub-optimal temperatures in temperate zones [30]. With this in mind, we considered that our investigation would be particularly meaningful if carried out under naturally-occurring conditions. The objective of this work was to investigate the gene expression profile of soybean leaves, under stressed and non-stressed conditions, after foliar application of the LCO NodBj-V (C_18∶1_, MeFuc) using Affymetrix GeneChip® analysis to screen a large number of soybean transcripts, with a particular focus on genes involved in stress regulation. Thus, we have examined changes in the patterns of gene expression, particularly stress response related genes, of plants already under stressful conditions.

## Results

We have studied the effects of LCO from *B. japonicum* strain 532C on the gene expression profile of soybean leaves following spray application at a sub-optimal growth temperature (15°C), corresponding to typical spring field conditions of eastern Canada.

**Figure 1:**
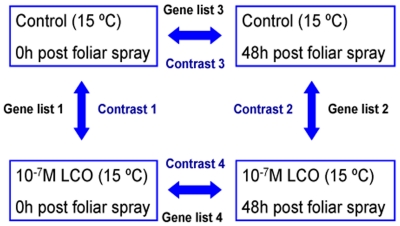
The four gene lists of interest in the microarray experiment, generated from four pair-wise comparisons (or contrasts) of gene expression profiles. 1) gene list 1 is the collection of genes differentially expressed between the dH2O control and 10−7 M LCO-treated plants, for leaves harvested 0 h after foliar spray; 2) gene list 2 is a list of genes differentially expressed between the dH_2_O control and 10^−7^ M LCO-treated plants, for leaves harvested 48 h after foliar spray; 3) gene list 3 includes all the differentially expressed genes of the dH_2_O) control plants during the 48 h time period after foliar spray; 4) gene list 4 consists of genes in the 10^−7^ M LCO-treated plants that were differentially expressed during the 48 h time period after spray treatment.

The first trifoliate leaves of the control and LCO-treated soybean plants (cv. OAC Bayfield) plants were then sprayed with dH_2_O or 10^−7^ M LCO, respectively, and harvested at 0 and 48 h after foliar spray. The four gene lists of interest in the microarray experiment were generated from four pair-wise comparisons (or contrasts) of gene expression profiles (Fig. 1); i.e., gene list 1 was the collection of genes differentially expressed between the dH_2_O-treated control and 10^−7^ M LCO-treated plants, for leaves harvested immediately (0 h) after the spray treatment; whereas gene list 2 was the list of genes differentially expressed between the dH_2_O-treated control and 10^−7^ M LCO-treated plants, for leaves harvested after 48 h of foliar spray. Similarly, the gene list 3 included all the differentially expressed genes of the dH_2_O-treated control plants 48 h after foliar spray and the gene list 4 was consisted of those genes that were differentially expressed in the 10^−7^ M LCO-treated plants 48 h after spray treatment.

### Effects of LCO under cold stress on gene expression

**Figure 2:**
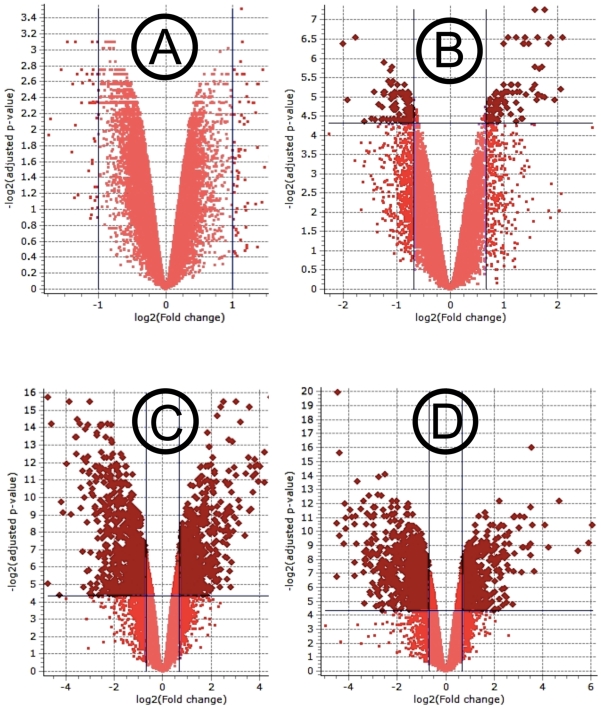
Volcano plots of the EB (Roke algorithm). A) contrast 1 (time = o h); B) contrast 2 (time = 48 h); C) contrast 3 (treatment  =  control); D) contrast 4 (treatment  =  LCO). The volcano plot is a graphical breakdown of the statistical analysis of microarray data. Each point in the plot corresponds to a statistically tested gene. The x-axis is the base 2 logaritm of the fold change, and the Y-axis is the negative base 2 logrithm of the q-value (or adjusted p- value). Thresholds for both the statistical significance (q≤0.05) and the biological significance are highlighted and assembled in the top left and top right corner of the graph, with exception of contrast 1 in which no hit was found.

**Figure 3:**
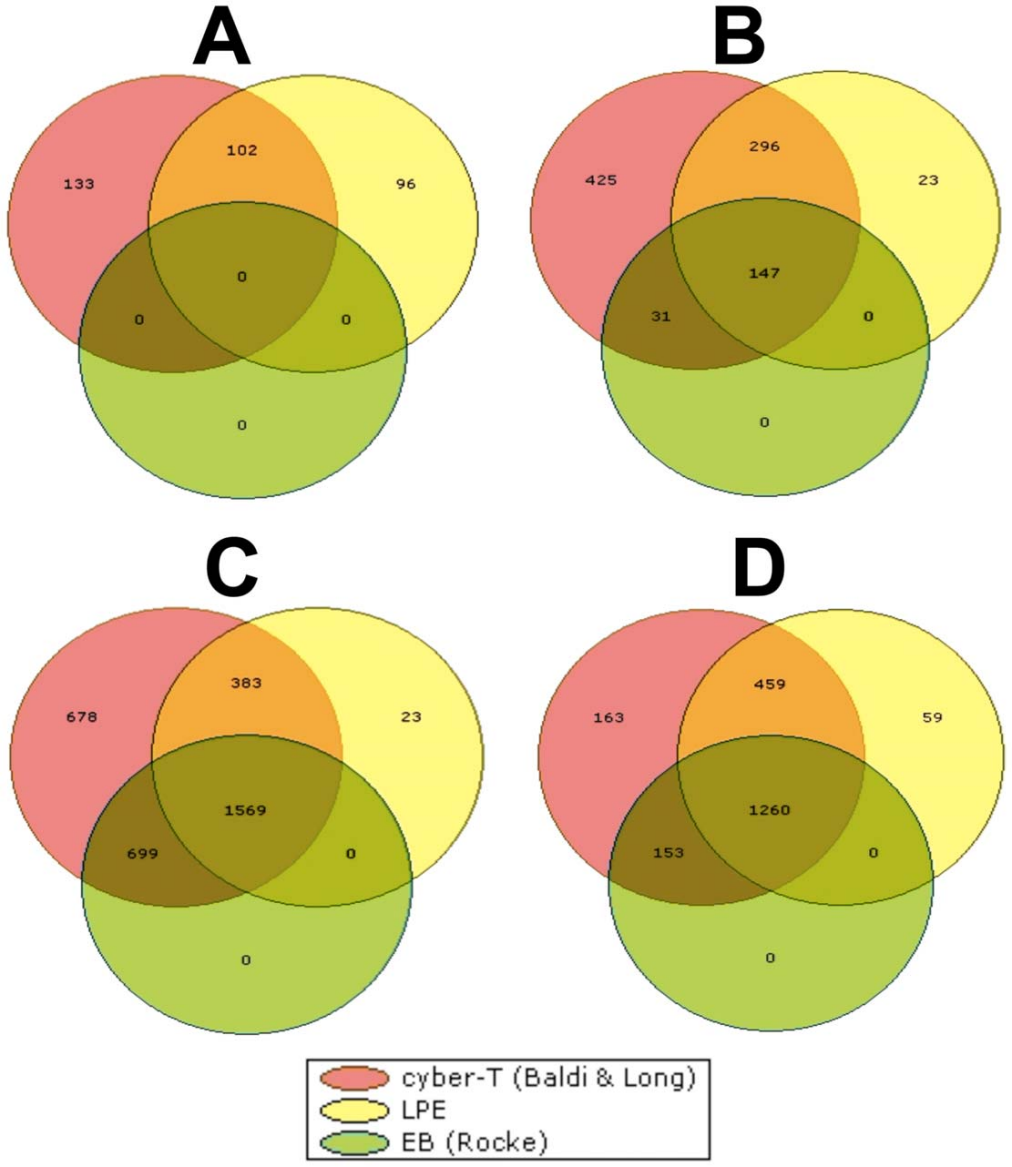
Venn diagrams of the output of differentially expressed genes, determined by Cyber-T, LPE and EB (Rocke) algorithms. A) contrast 1 (treatment  =  control vs. LCO, time = 0 h), the output is 0 genes; B) contrast 2 (treatment  =  control vs. LCO, time = 48 h), the output is 147 gene; C) contrast 3 (treatment  = control, time = 0 vs. 48 h), the output is 1569 genes; D) contrast 4 (treatment  =  LCO, time + 0 vs. 48 h), the output is 1260 genes.

All the genes in every gene list satisfied the criteria of statistical significance at q≤0.05 as determined by three statistical algorithms (Cyber-T, LPE and EB (Rocke), and biological significance at fold change ≥1.6. A Volcano plot of the EB (Rocke) algorithm of each gene list is given in Fig. 2, as a graphical breakdown of the two levels of significance. The number of differentially expressed genes in each gene list is displayed as Venn diagrams (Fig. 3). The stringency comparison of the three statistical algorithms is listed in Table 1. EB (Rocke) was more stringent than the other two algorithms, given that in three contrasts out of four, it yielded the smallest number of differentially expressed genes, as well as the lowest percentage of genes excluded in the final gene list (Fig. 3). All of the non-empty gene lists were annotated using Affymetrix annotation files obtained from SoyBase website. The functions of up- and down-regulated genes from each non-empty gene list were studied separately, through Gene Ontology (GO) information in the annotations. The genes were assigned to the following 11 functional classifications: 1) stress response; 2) signal transduction; 3) transcription; 4) protein metabolism; 5) transport; 6) developmental processes; 7) cell organization and biosynthesis; 8) electron transport; 9) other metabolism processes; 10) other functions; 11) function unknown. The four gene lists will be described in detail below. All microarray data were MIAME-compliant and were deposited in the Gene Expression Omnibus (GEO) database (http://www.ncbi.nlm.nih.gov/geo/) with the accession number of GSE20972.

**Table 1:** Stringency comparison of the three algorithms used in microarray data analysis.

		Cyber-T			LPE			EB (Rocke)		
Contrast	Output in the final gene list	Algorithm output	Excluded output	% of excluded output	Algorithm output	Excluded output	% of excluded output	Algorithm output	Excluded output	% of excluded output
**Contrast 1 (treatment = LCO, time = 0 h)**	0	235	235	100.00%	198	198	100.00%	0	0	NA
**Contrast 2 (treatment = LCO, time = 48 h)**	147	899	752	83.65%	466	319	68.45%	178	31	17.42%
**Contrast 3 (treatment = LCO, time = 0 h or 48 h**	1569	3329	1760	52.87%	1975	406	20.56%	2268	699	30.82%
**Contrast 4 (treatment = LCO, time = 0 h or 48 h)**	1260	2035	775	38.08%	1778	518	29.13%	1413	153	10.83%

The number of differentially expressed genes in the final gene list of each contrast is listed, together with the output of differentially expressed genes determined by each algorithm (Cyber-T, LPE and EB (Rocke)). For each algorithm, the number of genes excluded in the final gene list was calculated by subtracting the final gene list output from the algorithm input, and the percentage of excluded genes was listed and highlighted. EB (Rocke) was more stringent than the other two algorithms in that: 1) it yielded the smallest number of differentially expressed genes in all contrasts except contrast 3; 2) it yielded the lowest percentage of excluded genes in all contrasts, with the exception of contrast 3.

The gene list 1 (Fig. 1) i.e. differentially expressed genes resulting from comparison between the water control and 10^−7^ M LCO-treated plants harvested within the first hour after spray treatment showed that about 235 and 198 genes were differentially expressed by Cyber T and LPE algorithms, respectively. Interestingly, no differential gene expression was detected by the EB (Rocke) algorithm. Therefore, no gene was found significantly up- or down-regulated in this contrast as determined by all three statistical algorithms (Fig. 3A).

Gene list 2 (Fig. 1) contains genes differentially expressed between the water control and 10^−7^ M LCO-treated plants harvested 48 h after spray treatment. We detected 899, 466 and 178 differentially expressed genes by Cyber T, LPE and EB (Rocke) algorithms, respectively. A total of 147 genes were regarded as differentially expressed in this gene list as determined by all the three statistical algorithms (Fig. 3B).

**Figure 4:**
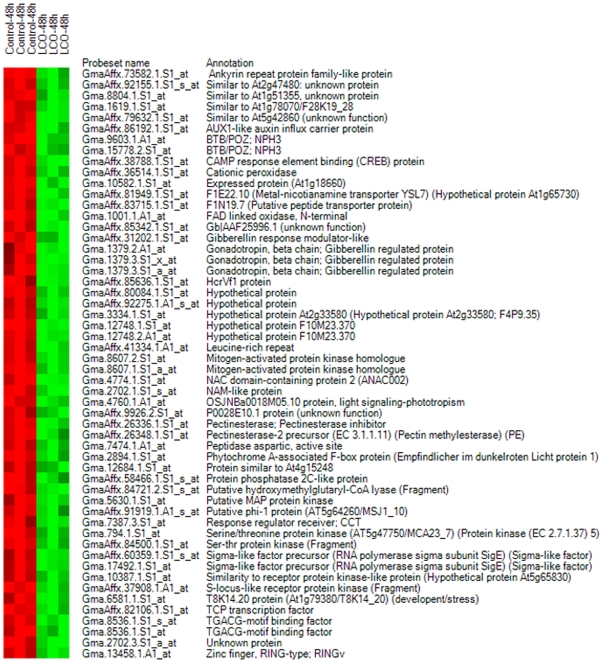
Heat maps of known genes in gene list 2 showing down-regulated genes. The heat map depicts the gene expression data of all replicates in a color scheme: red color represents up-regulation and green represents down-regulation; higher color brightness indicates a greater magnitude of differential expression and vice versa. Ideally, the same group of samples should have similar colors.

**Figure 5:**
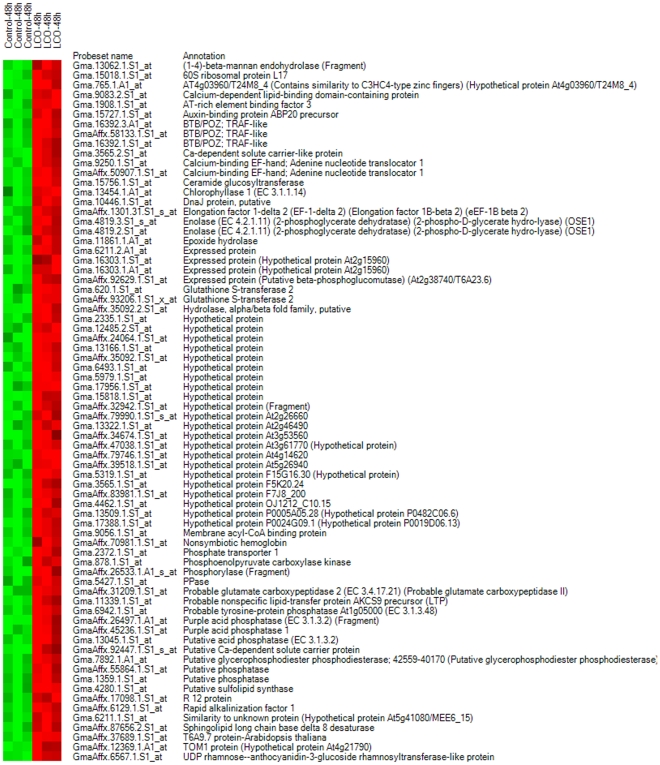
Heat maps of known genes in gene list 2 showing up-regulated genes. A heat map depicts the gene expression data of all replicates in a color scheme: red color represents up-regulation and green represents down-regulation; higher color brightness indicates a greater magnitude of differential expression and vice versa. Ideally, the same group of samples should have similar colors.

**Figure 6:**
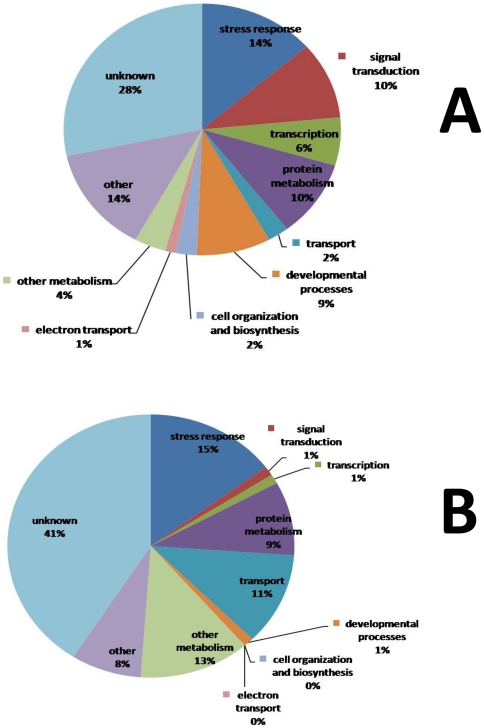
Functional classification of altered gene expression in contrast 2. Pie charts represent functional classification according to GO implications of A) down-regulated genes; B) up-regulated genes. Fourteen percent of the down-regulated genes and 15% of the up-regulated genes in contrast 2 were related to stress response. Sixteen percent of the down-regulated genes and 2% of the up-regulated genes were related to signaling (signal transduction and transcription). This indicates that under sub-optimal growth temperature (15°C), foliar spray of 10^−7^ M LCO induced the differential expression of stress related genes and components involved in signaling.

Analysis of gene list 1 led us to conclude that the experimental conditions along with the stringent statistical methods used showed no difference in the gene expression between the two samples, where as the differentially expressed genes identified in gene list 2 probably resulted from 10^−7^ M LCO foliar spray treatment at 15°C. Due to the very strict criteria for difference detection during data analysis (the selection of relevant genes was based on the overlap of the sets of genes whose expression levels were altered as detected by all 3 alogrithms used) it is possible that some genes responding to LCO treatment may have been missed. However, treated leaves with a material normally thought to be active when applied to roots and, because truly exceptional findings require exceptionally reliable proof. We have used a very cautious approach by using the same stringent statistical approach for all gene sets examined and conclude that the results are unbiased and reliable . Of the 147 differentially expressed genes in this list, we can say we a high degree of certainty that 65 were down-regulated and 82 were up-regulated. The gene list was annotated, and the gene names were determined according to the top hit in UniProt database. Heat maps were constructed separately for the down- (Fig. 4) and up-regulated genes (Fig. 5), and functional groups were assigned through GO implications (Fig. 6).

**Figure 7:**
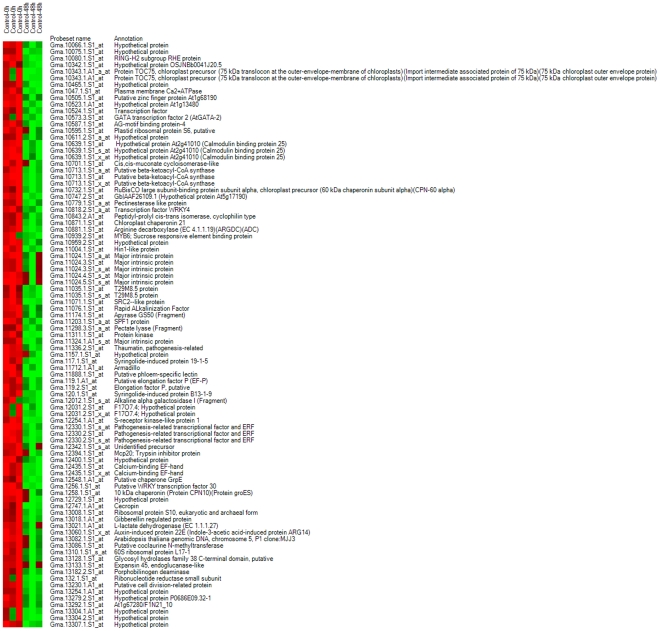
Heat maps of known genes in gene list 3, showing down-regulated genes.

**Figure 8:**
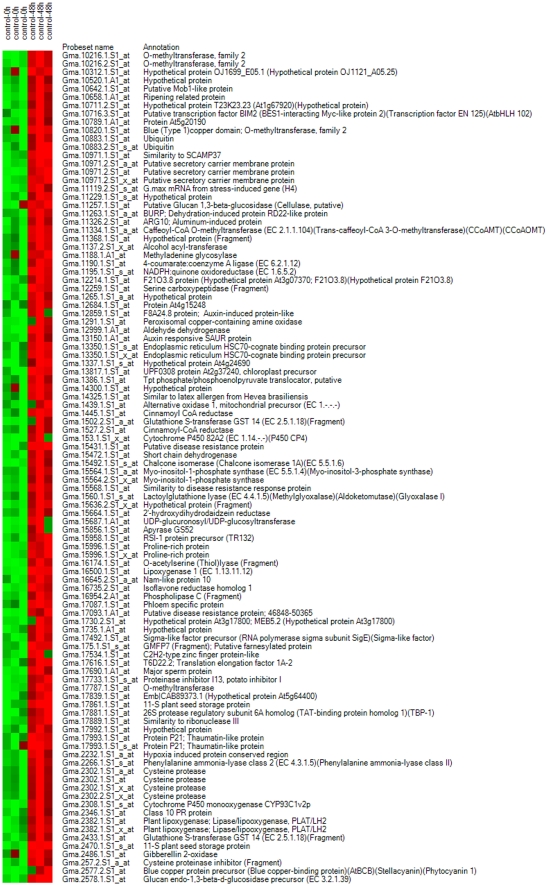
Heat maps of known genes in gene list 3, showing up-regulated genes.

**Figure 9:**
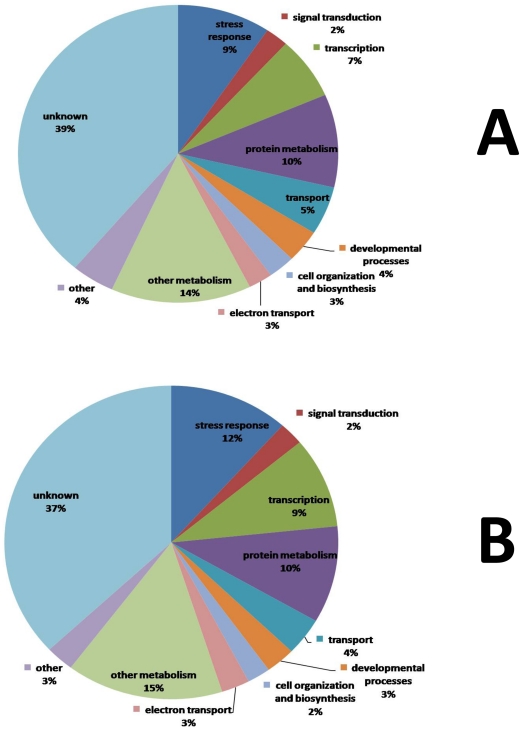
Functional classification of altered gene expression in contrast 3. Pie charts show functional classification according to GO implications of A) down-regulated genes; B) up-regulated genes. Nine percent of the down-regulated genes and 12% of the up-regulated genes in contrast 3 were related to stress response. Nine percent of the down-regulated genes and 11% of the up-regulated genes were related to signaling (signal transduction and transcription). This indicates that under sub-optimal growth temperature (15°C), the gene expression profile of soybean was dynamic over the period of 48 h; stress-related genes and signaling-related components were active during this time period.

Gene list 3 (Fig. 1) was constructed to show the collection of differentially expressed genes between the water control plants harvested at 0 and 48 h after spray treatment. Of these, 3329, 1975 and 2268 differentially expressed genes were detected by Cyber T, LPE and EB (Rocke) algorithms, respectively. A total of 1569 genes were regarded as differentially expressed in this gene list, as determined by all the three statistical algorithms (Fig. 3C), including 694 up-regulated genes and 875 down-regulated genes. Heat maps are given for down- (Figure 7) and up-regulated genes (Fig. 8), and functional groups were assigned through GO implications (Fig. 9).

**Figure 10:**
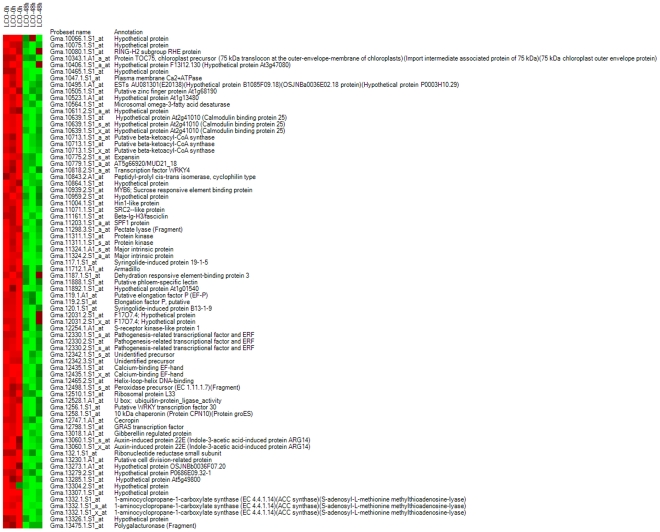
Heat maps of known genes in gene list 4 showing down-regulated genes.

**Figure 11:**
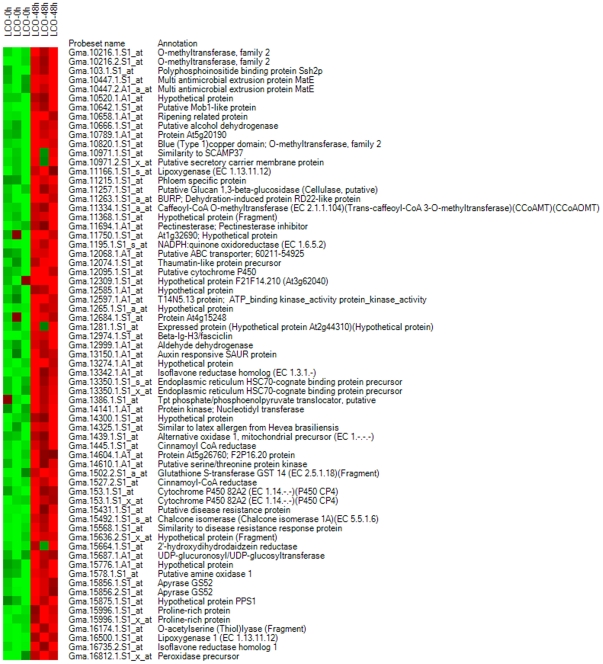
Heat maps of known genes in gene list 4 showing up-regulated genes.

**Figure 12:**
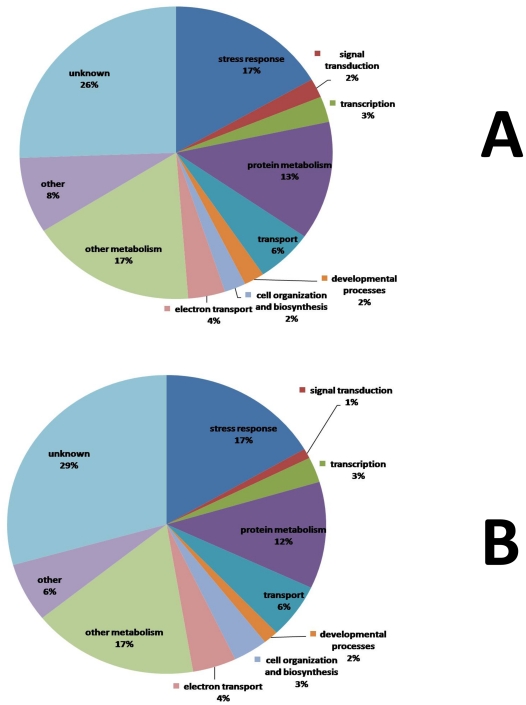
Functional classification of altered gene expression in contrast 4. Pie charts indicate functional classification according to GO implications of A) down-regulated genes; B) up-regulated genes. Seventeen percent of the down-regulated genes and 17% of the up-regulated genes in contrast 4 were related to stress response. Five percent of the down-regulated genes and 4% of the up-regulated genes were related to signaling (signal transduction and transcription). This indicates that under sub-optimal growth temperature (15°C), the gene expression profile of soybean was dynamic over the period of 48 h following the foliar spray of 10^−7^ M LCO; stress-related genes and signaling-related components were active during this time period.

Gene list 4 (Fig. 1) was the collection of genes differentially expressed between the 10^−7^ M LCO-treated plants harvested at 0 and 48 h after spray treatment. Of these, 2035, 1778 and 1413 were differentially expressed as detected by Cyber T, LPE and EB (Rocke) algorithms, respectively. A total of 1260 genes were regarded as differentially expressed in this gene list as determined by all the three statistical algorithms (Fig. 3D). In total, 1260 genes were differentially expressed, including 552 up-regulated genes and 708 down–regulated genes. Heat maps are given for the down- (Fig. 10) and up-regulated genes (Fig. 11), and functional groups were assigned through GO implications (Fig. 12).

### qPCR validation

**Figure 13:**
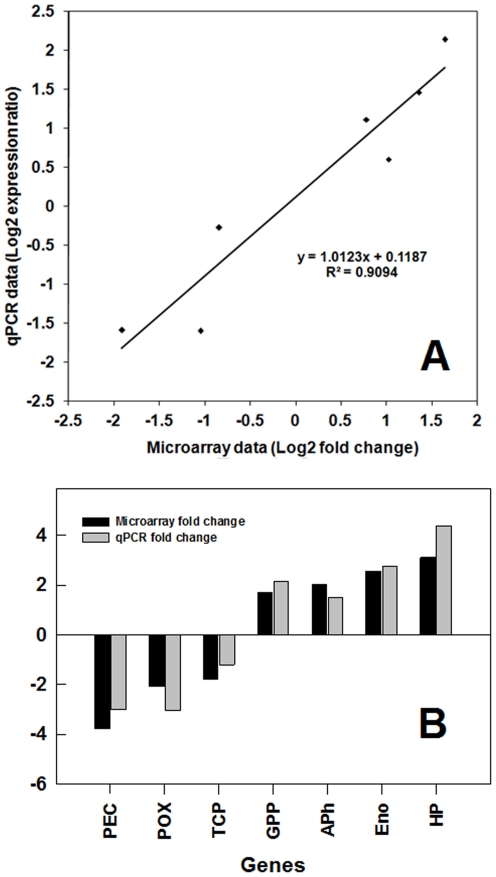
qPCR validation of the microarray data. Selection of target genes for qPCR validation, among the 147 differentially expressed soybean genes in gene list 2, using the random-stratified method. The 147 genes in gene list 2 were sorted in order of fold change, and divided into 7 equal-sized bins, each of which represented a stratum of fold change magnitude. From each stratum, one gene was randomly selected as a target gene for validation. The details of 7 selected genes in this study is shown in Table 2. A) Correlation of microarray data and qPCR data on a log scale; B) Correlation of microarray and qPCR data based on fold change.

In this study, the effects of LCO foliar application on soybean gene expression changes were of specific interest. Given that gene list 2 included most of the genes of interest (Table S1), 7 genes were selected from this gene list for qPCR validation using the random-stratified method (Table 2). The reliability of the microarray data was further tested by qPCR; in general, for the seven validated genes the microarray results were repeatable by qPCR at both statistical and biological significance levels (Fig. 13). For contrast 1, our qPCR results were in conformity with microarray data, where no significant differential gene expression was detected between microarray and qPCR results (Table 3). However, for contrast 2, a high correlation (r^2^ = 0.9) with high statistical significance was observed in fold change values of the qPCR and microarray data except, for only one gene. This indicated that qPCR validation is essential if one is to have a high degree of confidence in microarray data. The qPCR results showed a down-regulation of the *TCP* gene, like the microarray data but, unlike the microaaray data, this numerical difference was not statistically significant (Table 3).

**Table 2:** The 7 target genes selected for qPCR validation, plus the housekeeping reference gene *β-tubulin* [92].

Target Gene	Putative Function	GenBank ID	Primer Name	Primer Sequence	Tm (°C)	AmpliconSize (bp)	Amplicon Position
*ENO*	Pectinesterase-2 precursor (EC 3.1.1.11) (Pectin methylesterase) (PE)	AW597363.1	ENO-FENO-R	CACGAGGTTTAGCCCAAGATCAATTTGTCGCACCAAACAC	59 60	223	2–224 bp of AW597363.1
*HP*	Cationic peroxidase	BE346191.1	HP-FHP-R	CAAGCCTTGGTGGTATGCTTTGCATAGTTTCCAGCTTTCG	60 59	158	31–188 bp of BE346191.1
*PEC*	TCP transcription factor	BE474551.1	PEC-FPEC-R	GTGCAGGGCTCTTTCAGAACTGCTAGCACCTGCTCCTGTA	60 60	181	69–249 bp of BE474551.1
*POX*	Putative glycerophosphodiester phosphodiesterase; 42559–40170 (Putative glycerophosphodiester phosphodiesterase)	BE658341.1	POX-FPOX-R	AGAGTCCGTGGATCCTCCATACGAGATTGCCCTAGCACAG	61 60	157	284–440 bp of BE658341.1
*TCP*	Putative acid phosphatase (EC 3.1.3.2)	BI427245.1	TCP-FTCP-R	AGGTGGTTCTGCTGCGTATTAGAAGCAGATGATGGCCTGT	60 60	238	36–273 bp of BI427245.1
*GPP*	Enolase (EC 4.2.1.11) (2-phosphoglycerate dehydratase) (2-phospho-D-glycerate hydro-lyase) (OSE1)	BU550899.1	GPP-FGPP-R	CGGTTAGAGGTCCATTCCTGAGGGCACATGCTCATAACCT	60 60	188	194–381 bp of BU550899.1
*APH*	Hypothetical protein	CD401609.1	APH-FAPH-R	TGGCTTCGTTGCATGTAAAA TGGCCTCTCTTCCTAGTTCC	60 60	177	460–363 bp of CD401609.1
β *-tubulin*	Constituent of microtubules and binds to GTP	X60216.1	β*-tubulin* –Fβ*-tubulin* –R	TTGCAAGGGTTTCAAGTGTGATTGTAAGGCTCCACAACGG	58 60	165	479–643 bp of X60216.1

**Table 3:** Comparison and correlation between results obtained through qPCR and microarrray, in terms of fold change and p-value (or adjusted p-value).

Gene	Contrast 1				Contrast 2			
	Log2 (Fold change)		(Adjusted) p-value		Log2 (Fold change)		(Adjusted) p-value	
	qPCR	Microarray	qPCR	Microarray	qPCR	Microarray	qPCR	Microarray
APH	0.47	0.81	0.226	0.275	0.60	1.02	0.016*	0.035*
ENO	−0.33	0.72	0.404	0.549	1.46	1.36	0.001*	0.046*
GPP	−0.05	0.451	0.924	0.421	1.11	0.78	0.036*	0.040*
HP	0.65	1.37	0.170	0.293	2.14	1.64	0.001*	0.011*
PEC	−0.50	−1.39	0.102	0.168	−1.59	−1.91	0.001*	0.033*
POX	−0.04	−0.10	0.967	0.734	−1.604	−1.05	0.003*	0.030*
TCP	−0.24	−0.61	0.346	0.198	−0.28	−0.85	0.107	0.032*

Correlation coefficients of logarithm-scaled fold change derived from qPCR and microarray were calculated in order to determine result repeatability by the two different quantification approaches. Statistically significant results at p<0.05 are marked with asterisk. As no differentially expressed gene was detected by microarray in contrast 1, nor did qPCR yield any statistically significant results existed between the fold change results derived from both methods. In contast 2, a high level of correlation (R^2^ = 0.9) was found in the fold change values derived from qPCR and microarray, and 6 of the 7 genes (marked with asterisk) were regarded as statistically significant by both quantification methods.

## Discussion

The effects of LCO produced by *B. japonicum* strain 532C on the gene expression profile of soybean leaves, non-symbiotic tissue of the symbiotic host, by spray application under a sub-optimal growth temperature, a temperature typical of spring field conditions in eastern Canada was studied. Four gene lists were compiled based on the four pair-wise comparisons (or contrasts) of gene expression profiles i.e., list 1 is a set of genes differentially expressed in the dH_2_O control and 10^−7^ M LCO-treated plants when the leaves were harvested at 0 h after foliar spray; whereas list 2 contains the genes differentially expressed between the dH_2_O control and 10^−7^ M LCO-treated plants 48 h after the foliar spray. Gene list 3 contains all differentially expressed genes of the dH_2_0 control plants during the 48 h time period after foliar spray; similarly, list 4 comprised of differentially expressed genes in the 10^−7^ M LCO-treated plants 48 h after spray treatment.

No gene was found to be significantly up-regulated or down-regulated between the water control and 10^−7^ M LCO-treated plants (Gene list 1 and contrast 1) harvested immediately after spray treatment. Although, contrast 1 was excluded from further bioinformatics analysis due to the empty gene list, its importance should not be overlooked; it serves a number of important functions crucial to an accurate interpretation of the other three gene lists. First, the zero output of contrast 1 testifies to the rigor of the statistical methodology used in this study. While some studies in the literature have used only one statistical algorithm for microarray data analysis, such as Cyber-T or LPE [31], [32], three algorithms were used in this study, and only the overlapping output was deposited into the final gene list. While this data “trimming” procedure might lead to the discovery of fewer differentially expressed genes, it is necessary to minimze false positives and avoiding misleading data. Our evidence indicated that EB (Rocke) was the most stringent algorithm. As shown in Table 1, with the exception of contrast 3, EB (Rocke) yielded the smallest number of differentially expressed genes, and the lowest percentage excluded output. In contrast 1, a number of differentially expressed genes were identified by the Cyber-T and LPE statistical algorithms, but none was identified by EB (Rocke); on the other hand, although stringent, EB (Rocke) still had an exclusion percentage between 10 to 30% in the other three contrasts. The use of three algorithms instead of one has provided results that can be accepted with a high degree of confidence, avoiding data points that could otherwise be misleading. Further, the seven genes chosen from gene list 2 were successfully validated through the qPCR approach. Collectively, these lines of evidence demonstrate that the statistical method used for data analysis in this study was reliable and rigorous in distinguishing signal from noise, providing a reliable basis for the correct interpretation of all other results. In addition, the average CV for the data from control and LCO-treated plants harvested at 0 h were both below 10%. This argues that the absence of statistical significance in contrast 1 data was not a result of high variability. Furthermore, the zero output in gene list 1 indicated the absence of detectable very early signaling events triggered by 10^−7^ M LCO foliar spray. According to previous studies, subnanomolar concentrations of LCOs caused rapid electrophysiological changes within minutes on host root hairs and non-host cell suspension cultures [33], [34]. It is possible that the sub-optimal temperature desensitized soybean trifoliates, so that they responded to the LCO treatment slowly; lower growth temperatures are almost certain to result in slower responses in a non-homeotherm. Another explanation is that the early responses to LCOs are strictly physiological, where existing proteins were activated to pump ions across membranes, without any changes in gene expression activity. In fact, if differential gene expression were detected in contrast 1, it would have been uncertain as to whether this, or any other of the changes, resulted from background noise, experimental variability, error, or true effects triggered by LCO treatment. The zero output in contrast 1 eliminates all of these possibilities; therefore we can be affirmative regarding the stringency of statistical methods, uniformity of plants and experimental conditions, as well as the absence of early signaling events at the gene expression level.

A total of 482 down-regulated genes and 355 up-regulated genes were shared by contrasts 3 and 4; the overlapping genes account for 53% of the genes in list 3, and 66% of the genes in list 4, indicating that over half of the genes that were differentially expressed as the plants developed genes were common to both control and LCO-treated plants, although the magnitude of differential expression sometimes differed between the two groups. Functional categorization of these overlapping genes has indicated common transcription activities in the two groups of plants. The data indicate that plants in both contrasts were subject to maturation and aging, as reflected by a number of the gene activity changes, while adapting to the low temperature at the same time. There was also a significant number of overlapping components involved in both secondary metabolism and stress response. The overlapping gene lists of contrasts 3 and 4 also revealed the differential expression of an abundance of genes involved in various aspects of protein metabolism, such as protein phosphorylation, ubiquitination, glycosylation, folding, translation, and proteolysis, including a variety of differentially expressed protein kinases. These genes may be involved in signaling and transcription regulation.

After eliminating overlapping genes indicated by contrast 4, 393 down-regulated and 339 up-regulated genes were unique to contrast 3. Taken together with the overlapping genes described above, this gene list provides further information regarding gene expression activities unique to the control plants during the biological events occurring by 48 h after treatment, including aging, development, cold acclimation, and stress responses.

The differential expression of genes related to defence and abiotic stress at 15°C indicated by contrast 3 followed a similar temporal pattern to that of the overlapping gene list. The annotation for many of these genes were the same as that in the overlapping gene list, suggesting that the genes unique to gene list 3 were transcribed from different loci of the same gene family. After removal of overlapping genes demonstrated through contrast 3, 226 down-regulated and 197 up-regulated genes were unique to contrast 4. Genes in this list were differentially expressed, during the 48 h post treatment period, in response to LCO foliar spray. The presence of genes unique to contrast 4 suggests that apart from fine-tuning existing signaling pathways, LCO foliar application induces novel signaling pathways, which eventually leads to differential gene expression by 48 h after treatment, as indicated by contrast 2.

Genes encoding defence-related products unique to contrast 4 were detected, including the down-regulated defence resistance protein, as well as the up-regulated PAL, chitinase, multi-antimicrobial extrusion protein (MatE), and lipoxygenase. Accepted as a PR-protein, chitinase is represented by subfamilies of PR-3, 4 and 8 [33]. Chitinase can be induced by a variety of biotic and abiotic stress conditions, as well as SA, JA and ethylene; in addition to enhancing disease resistance during pathogen attack, chitinase suppresses host defence responses in plant-rhizobial and plant-mycorrhizal interactions; chitinase also plays roles in growth, embryogenesis and cold resistance [35]. It is very interesting that chitinase has been shown to accumulate under frost conditions and contributes to plant freezing tolerance [35]. The induction of chitinase genes in contrast 4 may have indicated interactions between LCO treatment and cold stress. Encoded by a large gene family, MatE proteins are transporters responsible for the detoxification of heavy metals and toxic secondary metabolites; they have also been shown to participate in SA-dependent defence signaling [36].

A number of genes associated with development and organization were found to be unique to contrast 4, such as the down-regulated expansin, together with the up-regulated histone H1, and NAC secondary cell wall thickening promoting factor (NST). Histone H1, a linker histone in eukaryotic chromatin, stabilizes chromatin higher-order structures [37]). It is noteworthy that as histone H1 exists in numerous isoforms, the differential expression of a few orthologue genes is not likely to cause any fluctuation in global histone H1 level [37]. *Arabidopsis* NSTs include the functionally-redundant NST1 and NST2, which are NAC-domain containing transcription factors regulating secondary cell wall thickening in various tissues [38]. Given the suppression of expansion and induction of NSTs, it is apparent that plants in contrast 4 had ceased expansion and started the synthesis of secondary cell walls. The same conclusion was drawn from contrast 3; nonetheless, in the control and LCO-treated plants, different gene loci were involved regarding secondary growth.

Similar to contrast 3, genes related to carbohydrate and lipid metabolism in contrast 4 were mostly down-regulated, while those involved in secondary metabolism were mainly up-regulated, such as flavonoid biosynthesis-related CHS and CHI, as well as the JA biosynthesis-related LOX, and secondary cell wall synthesis-related cellulose. As discussed above, many of these genes were members of the cytochrome P450 family. It is interesting that CHS, CHI and LOX are defence-related genes as well. The induction of these genes in contrast 4 may be related to the elicitor-nature of LCO, or cross-talk between secondary development and LCO-induced responses.

The data obtained from microarray analysis of soybean first trifoliate leaves sprayed with 10^−7^ M LCO at 15°C revealed the differential expression of 147 genes at 48 h after treatment, compared with the water control. Functional analysis of these genes led to the identification of genetic components associated with biotic/abiotic stress, signal transduction, development, primary and secondary metabolism, as well as transport. Of all the functional categories, unknown genes comprised the largest proportion (close to 30%), followed by stress-related genes (around 17%). A further breakdown of the stress-related genes led to the revelation of components involved in defense, oxidative stress, cold acclimation, hormonal response, MAPK cascade, phosphate deficiency and sulfolipid metabolism. Evidence also indicated probable cross-talk between LCO-induced response and cold acclimation.

### Effects of 10^−7^ M LCO foliar application on soybean gene expression at 15°C after 48 h

A total of 147 genes were differentially expressed 48 h after treatment with 10^−7^ M LCO foliar spray at 15°C, including 65 down-regulated genes and 82 up-regulated. The functional classification of these genes led to the discovery of components involved in a variety of biological functions; genes related to stress and signaling are of specific interest (for gene list 2 listed in Table S1). The putative products of the down-regulated genes included a cationic peroxidase, a protein phosphatase 2C (PP2C)-like protein, two homologues of mitogen-activated protein kinase (MAPK), a number of proteins related to auxin and gibberellin, proteins containing the NAC domain, a receptor kinase homologue, as well as a variety of transcription factors. The up-regulated genes encoded enolase, glutathione S-transferase, purple acid phosphatase, sulfolipid synthase, glutamate carboxypeptidase, chlorophyllase, as well as the defence-related TOM 1 and R 12 proteins.

### Antioxidant enzymes and plant hormones

Antioxidant genes were responsive to LCO foliar spray, notably the down-regulated cationic peroxidase and the up-regulated glutathione S-transferase (GST). Oxidative burst, referring to the rapid reactive oxygen species (ROS) generation and H_2_O_2_ accumulation, are commonly observed during early hypersensitive response following pathogen attack [39]. Plant peroxidases (POXs) are bifunctional enzymes involved in both the reduction of H_2_O_2_ and the production of ROS during oxidative burst [40]. Encoded by a large superfamily of genes, plant POXs are found in a variety of isoforms and interact with a broad range of substrates [40]. POXs play a variety of roles during the life cycle of plants, including roles related to stress (defense, oxidative stress and senescence); POXs are classified as PR-8 proteins [41]. POXs also participate in cell wall-related activities (lignification, suberization and cell wall protein cross-linking), plant hormone metabolism (auxin catabolism and ethylene biosynthesis), as well as development (somatic embryogenesis, growth regulation, light-mediated responses) [40], [42]. GSTs are glutathione (GSH)-dependent detoxifying enzymes abundant in plant tissues; encoded by at least 25 genes; GST proteins share as little as 10% similarity in amino acid composition [43]. Plant GSTs are highly responsive to pathogen attack, environmental stimuli and chemical treatment; GST level enhancement is commonly used as a marker for plant stress response [43]. Similar to POXs, GSTs are involved in development (cell division and senescence), and hormone metabolism (auxin and cytokinin) [43].

Most plant hormone-related components, mainly auxins and gibberellins, were down-regulated, such as AUX1-like auxin carrier protein, gibberellin regulated protein and gibberellic acid-intensive (GAI) protein. Mutation in the *Arabidopsis AUX1* gene led to the loss of root gravitropism, suggesting *AUX1* may encode a protein required for the hormonal regulation of plant root gravitropism [44]. GAI protein represses plant response to GA; this repression is reversed by exogenous GA [45].

### LCO-induced defense responses

Defense-related proteins were detected, such as the repressed HcrVf, and the induced TOM1 and R 12. The plant immune system is of two types, 1) transmembrane pattern recognition receptors acting in response to gradually evolving microbial- or pathogen-associated molecular patterns and 2) a response that is basically inside the cell, using the polymorphic NB-LRR protein products of R genes that are named after their characteristic nucleotide binding (NB) and leucine rich repeat (LRR) domains. Pathogen effectors can be recognized by NB-LRR proteins, and mediate matching defence responses. NB-LRR-mediated immune reponse is noticeable against pathogens that propogate on living host tissue, such as obligate biotrophs, or hemi-biotrophic pathogens, however it is not effective against necrotrophs i.e. pathogens that result in host tissue death following colonization [46]. In gene-for-gene resistance, plant resistance (R) protein confers resistance to a distinct pathogen by specifically recognizing the *avirulence* (*avr*) genes present in the pathogen. R proteins typically consist three functional domains: a leucine-rich-repeat (LRR) domain involved in pathogen recognition, a central nucleotide-binding site (NBS) functioning as a molecular switch, and an amino-terminal domain determining signaling specificity [47]. The *HcrVf* gene (homologue of the *Cladosporium fulvum* resistance genes of the *Vf* region) is similar to a gene that encodes a protein that promotes apple scab resistance [48]. Different from the previous two, TOM1 is not an R protein, but an integral plant membrane protein required for the propagation of tomato mosaic virus (ToMV); over expression of host TOM1 has been shown to inhibit ToMV multiplication [49].

### Possible cross-talk between cold stress and LCO-induced response

Understanding the cross talk and the mechanism of stress tolerance induced by LCO treatment by studying the genes involved will shed light on possible role of LCO in stress alleviation.

Two products of the cold-stress-related enolase gene were up-regulated by about 2-fold 48 h after LCO foliar spray under low temperature conditions, suggesting a possible interaction between cold response and LCO-induced response. Enolase, an enzyme known to participate in glycolysis, has shown to function in plant cold response and human programmed cell death (PCD) [50], [51]. The mutation of *LOS2*, a locus encoding a bi-functional enolase in *Arabidopsis*, impairs plant freezing and chilling resistance [50]. The involvement of enolase in primary metabolism, stress and senescence, as well as its responsiveness to LCO treatment under low temperature, indicates its possible protective role during stress conditions.

Further evidence suggests that there might be a cross-talk between cold response and LCO signaling. Two genes involved in sphingolipid metabolism were up-regulated, including sphingolipid long chain base Δ-8 desaturase and glucoceramide synthase, which may also be involved in the LCO-induced defense signaling. Ceramide is the core component of sphingolipid metabolic pathways. Interestingly, as the product of Δ-8 desaturase serves as the substrate of glucoceramide synthase, it was observed that Δ-8 desaturase activity is enhanced in the presence of glucoceramide [52], which explains why both genes were up-regulated in our study. Plant sphingolipids are involved in signal transduction, membrane stability, host-pathogen interactions and stress responses [52]. Sphingolipid confers membrane stability to plants, thus contributing to plant acclimation to drought and cold stress. It has also been found that cold-tolerant plants have higher levels of Δ-8 unsaturated sphingolipids, and lower levels of glucoceramides [52]. Thus in our case, the induction of these two genes may be an indication of cross-talk between cold and LCO signaling, but it cannot be determined whether this induction contributes to cold tolerance of the LCO-treated plants. In addition, fungal cerebrosides, which are glycosylated sphigolipids, are pathogenic elicitors leading to hypersensitive response, phytoalexin accumulation and cell death; this elicitation is dependent on the Δ-8 double bond [52]. Changes in the expression levels of many protein kinases and phosphatases were detected, including the down-regulated homologues of mitogen-activated protein kinase (MAPK) and protein phosphatase 2C (PP2C). The MAPK cascade is an important signal transduction pathway in plants, functioning in cell devision, differentiation, and hormonal response, as well as a variety of abiotic and biotic stresses; the cascade is a series of phosphorylation-dependent activations of MAPK components, starting with the activation of MAPK upon phosphorylation by MAPKK (MAPK kinase), which itself is activated by MAPKKK (MAPKK kinase) [53]. The regulation of MAPK cascades is intricate, as the various cascades form a complex network with cross-talk among the pathways [53]. Responses to abiotic stresses such as salt, cold, drought and wounding are mediated by overlapping MAPK cascades [54]. MAPKs are deactivated by protein phosphatases; PP2C is one of the major negative regulators of the MAPK cascades, especially during stress and ABA signaling [55]. The down-regulated MAPKs identified 48 h after LCO foliar spray under sub-optimal temperature may not be directly regulated by PP2C, since the latter was also down-regulated.

An NAC-domain containing protein and two NAM (no apical meristem) proteins were down-regulated 48 h after LCO exposure at 15°C. NAM, a member of the NAC family, was first characterized in petunia as a protein determining the position of meristems and meristem primordia; *nam* gene mutants usually die at the seedling stage due to failed shoot apical meristem development [56]. The NAC (NAM, ATAF1,2, CUC2) transcription factors are represented by a large gene family diverse in structure and function; in addition to embryonic, floral and vegetative development, auxin signaling and lateral root formation; members of the NAC family have been shown to regulate responses to pathogen attack and abiotic stress [57], [58]. NAC proteins are inducible by pathogen infection, wounding, cold shock, dehydration and high salinity; some NAC members are potentially involved in cross-talk with the ABA signaling pathways [57], [59]. Regulated at the transcriptional, post-transcriptional and post-translational levels, NAC proteins may participate in transcription factor networks and interact with other regulators of stress response [59].

Further evidence is supportive of LCO-induced stress response. Chlorophyllase (Chlase), and non-symbiotic hemoglobin, were up-regulated. Chlase catalyzes the initial step of chlorophyll breakdown during leaf senescence [60], [61]. Symbiotic plant hemoglobins are mainly present in root nodules, regulating oxygen supply to the N_2_-fixing rhizobia, whereas non-symbiotic hemoglobins are induced during hypoxic conditions, ensuring the oxygen supply for plant tissues, and detoxifying nitric oxide (NO) produced during hypoxia [62]. Non-symbiotic hemoglobins are also enhanced in active cells such as root tips and germinating seeds, possibly because rapidly growing cells are more likely to develop hypoxia [62]. The induction of these two genes indicates that LCO foliar spray may have led to hypoxic response and accelerated leaf senescence.

### Phosphate metabolism-related enzymes

Multiple copies of purple acid phosphatase (PAP) were up-regulated. Plant PAPs include the 35 kDa small PAPs and the 55 kDa large PAPs; PAP activity is greatly enhanced under phosphate deficient conditions, hydrolyzing both endogenous and exogenous phosphate storage compounds to ensure the phosphate supply [63]. In addition, mammalian and plant small PAPs have peroxidase activity, suggesting their involvement in ROS removal during oxidative stress [63].

Other phosphate metabolism-related enzymes were also up-regulated in the study, including glycerophosphoryl diester phosphodiesterase (GPX-PDE), and sulfolipid synthase. Plant glycerophosphodiesters, which accumulate as a catabolism product of phospholipid during membrane turnover and degradation, are hydrolyzed by GPX-PDE, releasing glycerol phosphate and the corresponding alcohol [64], [65]. Although the physiological roles of plant GPX-PDE are largely unclear, their accumulation during phosphate deficiency indicates that it may be responsible for releasing phosphate from soil or internal phospholipids [65]. Multiple copies of sulfolipid synthase homologues were up-regulated by at least 2-fold. Being an anionic natural surfactant molecule, sulfolipid is an important structural lipid of the plant photosynthetic membrane; *Arabidopsis* sulfolipd synthase is encoded by *SQD* genes, and mutation in *SQD* genes impaired plant adaptation to phosphate deficiency [66], [67]. This indicates that sulfolipid biosynthesis acts to compensate for the insufficient anionic phospholipid production when phosphate becomes a limiting macronutrient [66]. Interestingly, apart from phosphate deficiency, sulfolipids accumulate when plants are exposed to salt, drought and cold stress [67].

The up-regulation of phosphoenolpyruvate carboxylase (PEPC) kinase is of specific interest, as the multi-function enzyme is involved in primary metabolism, symbiosis, as well as salt stress response. PEPC is the enzyme catalyzing the primary CO_2_ fixation step in C_4_ and crassulacean acid (CAM) plants; in C_3_ plants, PEPC replenishes the tricarboxylic acid cycle with intermediates [68], [69]. In legume root nodules, PEPC is essential for the synthesis of C_4_ dicarboxylate, the carbon skeleton of amino acids assimilated by the bacteroids [68]. In addition, PEPC plays roles in fruit ripening, regulating cellular pH, and providing malate to guard cells [69]. PEPC kinase is a Ca^2+^-independent serine/threonine kinase that phosphorylates PEPC; strikingly, PEPC kinase activity was enhanced by salt stress in *Sorghum vulgare*; further analysis revealed that the induction was related to ion toxicity rather than osmotic stress [70]. Although it is uncertain which aspect of PEPC function caused its responsiveness to LCO foliar spray, any of the three indicated could be affected.

### Transport and development

LCO foliar spray may also have enhanced mitochondrial metabolite exchange, as a number of mitochondrial carrier family (MCF) homologues were up-regulated. Plant mitochondria serve in a variety of functions, including respiration, photorespiration, photosynthesis in C_4_ and CAM plants, as well as catabolism of storage compounds during seed germination; there is therefore a need for a mitochondrial transporter system in order to exchange metabolites between the mitochondria and the cytosol [71]. MCFs are transmembrane proteins with three conservative domains, each of which consists two transmembrane α-helices and a hydrophilic extramembrane loop; however, the physiological roles of most MCF proteins are poorly understood, partially due to the low abundance of transmembrane proteins and technical difficulties in studying them [71], [72].

A rapid alkalinisation factor (RALF) was induced. The abundance of RALF homologues in plants indicates a fundamental function, however, the nature of this function is not yet well known [73]–[75]. Although extracellular alkalinization is a phenomenon that commonly occurs during pathogen attack and environmental stimuli, a study in poplar cell culture demonstrated that the suppression of RALF by MeJa was more likely to be development-related than stress-related [73].

Glutamate carboxypeptidase (GCP) II was found to be up-regulated in contrast 2. GCP is encoded by the *AMP 1* (*ALTERED MERISTEM PROGRAM 1*) gene in *Arabidopsis*; functional studies through mutational analysis revealed its involvement in the development of shoot apical meristems, flowering, photomorphogenesis, and cytokinin biosynthesis [76], [77].

### Transcription factors

A variety of transcription factors and other signaling-related components were detected. A sigma E-like factor (sigE), which is a component of the prokaryotic RNA polymerase that specifically recognizes and binds to the gene promoter region [78], was down-regulated. Plant sigE is encoded by both plastid and nuclear genes, and plays an important role in maintaining the RNA polymerase function [79]. The BTB/POPZ-domain containing proteins were differentially expressed, three copies of which were induced while two were suppressed. The BTB (Broad Complex, tramtrack and bric à brac) or POZ (poxvirus and zinc finger) is a conserved domain found at the NH_2_-terminal of zinc fingers, poxvirus and actin-binding proteins; it is a protein-protein interaction motif involved in the organization of macromolecular complexes [80]. Strikingly, the two suppressed copies of BTB/POZ protein were also homologous to the phototropic-response protein NPH3 (nonphototropic hypocotyl 3), which is a UV-A/blue light receptor inducing the phototropic response [81].

### qPCR validation of microarray results

Our results indicated that in general, microarray data patterns were reproducible when qPCR was performed at both statistical and biological significance levels and all the 7 selected target genes showed comparable expression patterns demonstrated by two methodologies.

In agreement with our Microarray data, for contrast 1, where no differential gene expression was detected by microarray, our qPCR results were not statistically significant (Table 3). Since most of the qPCR p-values were higher than 0.3, it is reasonable to assume that the expression levels of these genes were not affected by LCO treatment at 0 h. However, our results also indicate that qPCR validation is still necessary for microarray discoveries, as some minor disagreement in statistical significance was still found for one of the 7 validated genes. The qPCR results revealed a down-regulation of the *TCP* gene by 1.2 fold, but it was not statistically significant. A number of factors might have contributed to this disagreement. First, the amplification-based qPCR method is more sensitive and accurate in gene quantification, while the hybridization-based microarray technique is more powerful in large-scale gene expression analysis [82], [83]. Also, only 3 biological replicates were used for microarray analysis in this study, while 6 biological replicates were used for qPCR validation. Thus, it is reasonable that the fold change and significance levels derived from the two methods were similar but not identical. Further, the TCP gene encodes a transcription factor generally expressed at low levels. It is suggested in other studies that accurate quantification by microarray is challenged by low transcript abundance, such as in the case of transcription factors; the low hybridization signal of rare transcripts is often treated as background noise, while lowering the detection threshold leads to higher rates of false positives [25], [26], [84]. High throughput qPCR profiling was shown to be a better alternative to quantify transcription factors [82]. As indicated by previous studies, more emphasis should be placed on the direction of gene expression change indicated by microarray, rather than the magnitude of change [85].

Although previous studies have shown that LCO foliar spray may trigger differential expression of stress-related genes in the absence of external stress, this study revealed that in the presence of external stress (low temperature), LCO foliar spray also caused differential expression of stress-related genes, possibly through re-programming of host stress response. In addition to the analysis carried out at 48 h after treatment, a temporal analysis of control and LCO-treated plants during the 48 h following foliar spray was performed, which had led to the identification of thousands of differentially expressed genes in both treatment-groups. Over half of temporally altered genes in the control and LCO-treated plants were overlapping. Both the overlapping and treatment-specific genes were functionally categorized. Similar to findings at the 48 h time point, stress-related genes occupied the second largest percentage (around 17%), after unknown genes (around 30%). These stress-related genes are known to be involved in defence, cold acclimation, hormonal response, transcription regulation, and secondary metabolism. Notably, cold acclimation-related genes were mostly down-regulated, along with components responsible for carbohydrate and lipid metabolism; while genes related to the biosynthesis of secondary metabolites such as flavonoids and lignin were mainly up-regulated. The data indicate that plants in both treatment-groups were subject to maturation and aging, while adapting to the low temperature stress conditions at the same time. The data also suggest that under sub-optimal temperature, soybean plants respond to LCO foliar spray by both re-programming existing signaling pathways and activating novel pathways.

To summarize, the genes differentially expressed as a result of LCO foliar spray under sub-optimal temperature, were those related to enhancement of various stress resistances, senescence and development-related genes, especially those involved in defence, cold, oxidative stress, and phosphate deficiency. It is also interesting that most plant hormone-related genes were down-regulated, together with the MAPK-related components. This suggests that LCO can be perceived by an unknown receptor(s) in non-symbiotic tissues under sub-optimal temperature, leading to the reprogramming of plant stress responses.

## Materials and Methods

### Approach to the data

We exerted strict criteria at every stage, from data collection to statistical analysis. Total RNA was extracted, and the quality was monitored to ensure the quality and consistency. The transcript abundance profiles in the soybean trifoliates were analyzed prior to all data analysis. The microarray raw data were observed in the form of intensity maps to ensure that the data points were positioned similarly to the physical array, and the color scale corresponded to the signal intensities. Our results showed no obvious defect in the intensity maps of any of the 12 arrays we performed, indicating good physical conditions of all the arrays used in this study. The expression data was normalized using the RMA algorithm. The microarray experiment in this study was designed in a circular manner, comprising 4 gene lists generated by 4 contrasts of biologically relevant samples (Fig. 1) We performed Principal Component Analysis (PCA) on the normalized arrays which simplify data sets by capturing the major sources of variance in the study, leading to reduced dimensions, indicating that the biological replicates were uniform in nature and responded similarly to the same treatment.

### LCO purification via HPLC

*Bradyrhizobium japonicum* strain 532C was grown in Yeast Extract Mannitol (YEM) broth medium (10 g mannitol, 0.5 g K_2_HPO_4_, 0.1 g MgSO_4_, 0.2 g MgSO_4_•7H_2_O, 0.1 g NaCl, 0.4 g yeast extract, 1 L dH_2_O, 15 g agar) at 28°C until the cell density, as determined from OD_600_ values, reached 4×10^8^ cells per mL. The rhizobia were sub-cultured in fresh YEM medium, until they reached the exponential growth phase (4 days). To induce LCO production, genistein (Sigma, St. Louis, USA) stock solution in 100% methanol was added into the medium to a final concentration of 5 µM. The culture was incubated for another 2 days to allow sufficient LCO production.

Extraction was started by using phase partitioning against 40% HPLC-grade n-butanol while shaking for 30 min at 150 rpm. The organic fraction was collected and evaporated at 50°C in a rotary evaporator (Yamato, NJ, USA) under vacuum. The brown and viscous remaining material was redissolved in 18% acetonitrile, loaded onto a C18 column (PRESEP™ Fisher Scientific, Montreal, QC, Canada), and eluted three times with 10 mL of 30% acetonitrile. Another elution was performed with 10 mL of 60% acetonitrile. This eluent contained the LCO, and was further fractionated by HPLC, with Waters 501 pumps and a Waters 401 detector set at 214 nm, and a WISP712 autosampler using a C18 reverse phase column (Vydac, CA, USA). The chromatography was conducted for 60 min with an acetonitrile linear gradient from 18 to 60%. The peak displaying the same retention time as that of an LCO standard (NodBj-V (C_18∶1_, MeFuc)) from *B. japonium* strain 532C was identified as the LCO peak of interest. The eluent containing this peak was collected, freeze-dried, rechromatographed and the peak collected. The LCO concentration was determined by measuring the area under the HPLC peak of a standard of a known concentration, and comparing it with the peak of the purified LCO sample.

### Plant growth, treatment and harvest

The soybean cultivar (cv.) used in this study was OAC Bayfield. The seeds were first surface-sterilized with 25% commercial bleach for 2 min, and rinsed five times with distilled water (dH_2_O). Sterilized seeds were scattered evenly on a 5 cm thick bed of autoclaved vermiculite (Holiday®, Normiska Co.), covered with another 1 cm layer of vermiculite, and watered thoroughly with dH_2_O to allow germination in a growth chamber. The chamber temperature was maintained at a constant 25°C with a 16 h photoperiod and an 8 h period of darkness. On the 7^th^ day after sowing (DAS), uniform V1-stage seedlings [86] were selected and transferred to 10 cm pots containing a 1∶1 v/v mixture of autoclaved sand and turface, and grown under the conditions described above. At 12 DAS, V2-stage seedlings were transferred to a different growth chamber and kept at 15°C, with a 16 h photoperiod and a dark period of 8 h. After acclimatizing at 15°C for 5 days, plants were ready for treatment and harvest. The plants were watered with ½ strength Hoagland's solution [87] at the same time every day from transplant to harvest.

At 17 DAS, the V3-stage soybean plants were divided into groups, each group representing a treatment and sampling time. The first trifoliolate leaf of each plant was sprayed with 2 mL of sterilized dH_2_O containing one of the following treatments:

1. **Control**: 0.02% Tween® 20
2. **10^−7^ M LCO**: 10^−7^ M LCO and 0.02% Tween® 20

The sprayed trifoliolate leaves of each plant were harvested at 0 (within 20 min of spray application) and 48 h after treatment. The leaves were rapidly cut off from the stem with a scalpel, wrapped individually in aluminum foil and immediately submerged in liquid N_2_. All samples were stored at −80°C until extraction.

### RNA extraction, array labeling, hybridization and scanning

Frozen leaf tissue was rapidly ground in liquid N_2_, and total RNA was extracted from ∼80 mg of finely-ground, frozen tissue using the Qiagen RNeasy® Plant Mini Kit (Qiagen, Missisauga, ON, Canada), following the protocol provided by the manufacturer. Following RNA extraction, the concentration of total isolated RNA was measured using a NanoDrop® ND-1000 spectrophotometer (NanoDrop Technologies, Wilmington, DE, USA). All samples were stored at −80°C until use.

Total RNA was isolated as described above, and adjusted to a concentration of 250 ng µL^−1^. Transcript abundance was measured using GeneChip® Soybean Genome Arrays (Affymetrix, Santa Clara, CA, USA). RNA quality was determined using a 2100 Bioanalyzer (Agilent Technologies, Santa Clara, CA, USA). All samples were of good quality and 3.5 µg RNA was used to synthesize labeled target cRNA using the GeneChip® HT One-Cycle Target Labeling and Control kit (Affymetrix). Fifteen micrograms of fragmented cRNA were hybridized overnight with the chip, followed by staining and washes using the GeneChip® Fluidics Station 450 robot (Affymetrix). The microarray chips were scanned with a GeneChip® Scanner 3000 7 G (Affymetrix) for fluorescence intensity profile.

### Microarray experimental design and data analysis

For each treatment and each sampling time, a total of 6 plants were harvested for RNA extraction. Half of those samples were used for microarray hybridization, while all of them were used for qPCR validation. The microarray experiment was structured following a Completely Randomized Design (CRD) with 3 replicates per treatment per sampling time. After foliar spray treatment, each plant was randomly positioned in the growth chamber. The presence of 2 treatment conditions (dH_2_O control and 10^−7^ M LCO) and 2 sampling times (0 and 48 h post treatment) led to the generation of 4 gene lists in this study. Each gene list was generated from a pair-wise comparison of gene expression profiles between relevant groups of plants.

All calculations were performed by FlexArray software [88], version 1.4.1. The robust multi-array average (RMA) algorithm [89] was utilized to normalize the microarray raw data; this corrects the background by subtracting estimates derived from the lowest signals, in order to enforce a common probe distribution across all 12 arrays used. After RMA normalization, the median log-scale expression measure of each GeneChip® was zero, as the base 2 logarithm of RMA signals were median-centered. Three separate algorithms were used to calculate p-values: Cyber-T, Local Pooled Error (LPE) and Empirical Bayes (EB; Rocke). For each algorithm, the False Discovery Rate (FDR) correction procedure was used, with the Benjamini Hochberg algorithm. As a result, an adjusted p-value (*q*-value) was produced for each comparison. The corrected data was then filtered with two criteria: data points with *q*-values of ≤0.05 were considered statistically significant, and genes expressed at fold-changes of ≥1.6 or ≤−1.6 were considered biologically significant. A gene list was created by combining all data points that qualified based on the two significance criteria for all 3 algorithms. Genes from that list were further examined for biological relevance in the context of the treatment conditions. The derived gene lists were annotated using the Affymetrix GeneChip® Soybean Genome Array Annotation, obtained from the SoyBase website [90]. Functional implications of the genes in the lists were specified in Gene Ontology (GO) information contained in the annotation file. Gene functions were classified into groups according to the GO biological process information.

### DNase treatment and cDNA generation

For qPCR validation, the same batch of RNA samples was used as for microarray hybridization. All samples were treated with Turbo™ RNase-free DNase (Ambion Inc., Applied Biosystems, Streetsville, ON, Canada) prior to reverse-transcription (RT), in order to remove any contaminating genomic DNA (gDNA) that would interfere with the accuracy and specificity of qPCR validation. From each sample, 1 µg of RNA was used with oligo-dT primers (Invitrogen, Burlington, ON, Canada) to construct cDNA via RT reaction using the OmniScript® Reverse Transcription Kit (Qiagen), following the manufacturer's instructions. After the reaction, all cDNA samples were stored at −20°C until further use.

### Real-time qRT-PCR

A total of 7 genes were selected from the gene list of interest for qPCR validation, using the random-stratified method [91]. The housekeeping soybean *β-tubulin* gene was used as the reference gene for relative quantification [92]. Target gene-specific primers for qPCR were designed using Primer3 on-line software [93], version 0.4.0.

Quantitative real-time PCR was performed using the Stratagene Mx3000P Real-Time PCR System (Agilent Technologies, Santa Clara, CA, USA). Each reaction contained 12.5 µL of 2× Stratagene Brilliant® SYBR® Green QPCR Master Mix (Agilent Technologies), 0.375 µL of freshly diluted reference dye (1∶500 v/v), optimized volumes of sample cDNA, the forward and reverse primers of one target or reference gene, and a variable volume of nuclease-free PCR-grade water for a final volume of 25 µL. The reactions were performed in 0.2 mL volume strip tubes and sealed with flat optical caps (Stratagene, Agilent Technologies). The amplification for the *β-tubulin* gene consisted of a 10 min incubation at 95°C to activate the Taq DNA polymerase, 45 cycles of 30 s at 95°C, 1 min at 55°C, and 30 s at 72°C followed by dissociation curve analysis over a 40°C temperature gradient at 0.066°C s^−1^ from 55 to 95°C. The amplification of the other target genes followed the same procedure as above, but was performed at specific annealing temperatures, as optimized beforehand. The specificity of amplification was confirmed by a single melting point on the dissociation curve, a single band on 2% agarose gel, as well as the sequencing results (Sequencing Platform, Génome Québec and McGill University Innovation Centre).

Transcript levels for the target genes were determined by an automatic comparison of individual cycle threshold (Ct) values with the serial dilution qPCR standard curve for each specific gene. The qPCR efficiency was also reflected in the standard curves, which ranged from 85 to 100%. Relative gene expression was calculated using the Relative Expression Software Tool (REST; [94], [95]), version 2008_2.0.7, which used the following equation to calculate the ratio of gene expression:
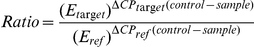



In this equation, E_target_ and E_ref_ refer to the qPCR amplification efficiencies of the target gene and the reference gene respectively, as indicated by the standard curves; whereas CP, referring to the cross point, is equivalent to the Ct value [95]. A gene was determined to be significantly up-regulated or down-regulated, relative to the control treatment, at p≤0.05.

## Supporting Information

Table S1 **List of genes related to stress and signal transduction in gene list 2.** The putative products ofthe down-regulated genes included a cationic peroxidase, a protein phosphatase 2C (PP2C)-like protein, two homologues of mitogen-activated protein kinase (MAPK), a number of proteins related to auxin and gibberellin, proteins containing the NAC domain, a receptor kinase homologue, as well as a variety of transcription factors. The up-regulated genes encoded enolase, glutathione S-transferase, purple acid phosphatase, sulfolipid synthase, glutamate carboxypeptidase, and chlorophyllase, as well as defence-related TOM 1 and R 12 proteins.
(DOCX)
