# Supplementary material for: Changes in Soybean Global Gene Expression after Application of Lipo-Chitooligosaccharide from Bradyrhizobium japonicum under Sub-Optimal Temperature
Source: PLoS One. 2012 Feb 13;7(2):e31571. doi: 10.1371/journal.pone.0031571 (PMC3278468; doi:10.1371/journal.pone.0031571)
Supplement: Table S1 — List of genes related to stress and signal transduction in gene list 2. The putative products ofthe down-regulated genes included a cationic peroxidase, a protein phosphatase 2C (PP2C)-like protein, two homologues of mitogen-activated protein kinase (MAPK), a number of proteins related to auxin and gibberellin, proteins containing the NAC domain, a receptor kinase homologue, as well as a variety of transcription factors. The up-regulated genes encoded enolase, glutathione S-transferase, purple acid phosphatase, sulfolipid synthase, glutamate carboxypeptidase, and chlorophyllase, as well as defence-related TOM 1 and R 12 proteins. (DOCX) [file pone.0031571.s001.docx]

Table S1. List of genes related to stress and signal transduction in gene list 2. The putative products ofthe down-regulated genes included a cationic peroxidase, a protein phosphatase 2C (PP2C)-like protein, two homologues of mitogen-activated protein kinase (MAPK), a number of proteins related to auxin and gibberellin, proteins containing the NAC domain, a receptor kinase homologue, as well as a variety of transcription factors. The up-regulated genes encoded enolase, glutathione S-transferase, purple acid phosphatase, sulfolipid synthase, glutamate carboxypeptidase, and chlorophyllase, as well as defence-related TOM 1 and R 12 proteins.

| **Probeset** | **GenBank**  **ID** | **Uniprot**  **Top Hit** | **Fold**  **change** | **Q-value** | **Putative function** | |  |
| --- | --- | --- | --- | --- | --- | --- | --- |
| GmaAffx.86192.1.S1_at | BU550323 | Q76DT1 | -1.76 | 0.04 | AUX1-like auxin influx carrier protein | |  |
| GmaAffx.36514.1.S1_at | BE658341 | Q41324 | -2.07 | 0.03 | Cationic peroxidise | |  |
| GmaAffx.31202.1.S1_at | BF008821 | Q9AS97 | -2.13 | 0.03 | Giberellin response modulator-like | |  |
| Gma.1379.2.A1_at | CD409927 | Q1S753 | -2.74 | 0.05 | Gonadotropin, betan chain; Gibberellin regulated protein | |  |
| Gma.1379.3.S1_x_at | BM527366 | Q1S753 | -2.41 | 0.04 | Gonadotropin, betan chain; Gibberellin regulated protein |  |  |
| Gma.1379.3.S1_a_at | BM527366 | Q1S753 | -2.35 | 0.03 | Gonadotropin, betan chain; Gibberellin regulated protein | | |
| GmaAffx.85636.1.S1 at | BM143123 | Q949G9 | -2.08 | 0.04 | HcrVfl protein | | |
| Gma.8607.2.S1_at | AW349101 | Q9XF36 | -2.22 | 0.02 | Mitogen-activated protein kinase homolgue | | |
| Gma.8607.1.S1_a_at | BE823324 | Q9XF36 | -1.83 | 0.03 | Mitogen-activated protein kinase homologue | | |
| Gma.4774.1.S1_at | AW310386 | Q39013 | -1.65 | 0.03 | NAC domain-containing protein 2 (ANAC002) | | |
| Gma.2702.3.S1_a_at | AW185294 | Q43521 | -1.91 | 0.03 | NAC-domain protein | | |
| Gma.2702.1.S1_s_at | BU762960 | Q9FY93 | -1.89 | 0.03 | NAM-like protein | | |
| Gma.4760.1.A1_at | AW310321 | Q7XQ98 | -2.14 | 0.03 | OSJNBa0018M05.10 protein | | |
| Gma.2894.1.S1_at | BG551463 | Q8LEA8 | -2.02 | 0.04 | Phytochrome A-associated F-box protein (Empfindlicher im dunkelroten Licht protein l) | | |
| GmaAffx.58466.1.S1_s_at | AW396505 | Q9FKX4 | -2.62 | 0.05 | Protein phosphatise 2C-like protein | | |
| GmaAffx.60359.1.S1_s_at | BM093489 | Q9ZNX9 | -2.35 | 0.03 | Sigma-like factor precursor (RNA polymerase sigma subunit SigE) (Sigma-like factor) | | |
| Gma.17492.1.S1 at | CD398389 | Q9ZNX9 | -2.30 | 0.03 | Sigma-like factor precursor (RNA polymerase sigma subunit SigE) (Sigma-like factor) | | |
| Gma.10387.1.S1_at | BG352903 | Q9FH86 | -2.05 | 0.05 | Similarity to receptor protein kinase-like protein (Hypothetical protein At5g65830) | | |
| GmaAffx.82106.1.S1_at | BI427245 | Q1SE50 | -1.80 | 0.03 | TCP transcription factor | | |
| Gma.85361.S1_s_at | L28003 | Q39895 | -2.34 | 0.02 | TGACG-motif binding factor | | |
| Gma.85361.S1_at | L28003 | Q39895 | -2.03 | 0.04 | TGACG-motif binding factor | | |
| Gma.13454.1.A1 at | CD416724 | Q8GTM4 | 2.37 | 0.05 | Chlorophyllase 1 (EC 3.1.1.14) | | |
| Gma.4819.3.S1_s_at | CA783243 | Q42971 | 1.84 | 0.05 | Enolase (EC 4.2.1.11) (2-phosphoglycerate dehydratase) (2-phospho-D-glycerate hydro-lyase) (OSE1) | | |
| Gma.4819.2.S1_at | AW597363 | Q42971 | 2.56 | 0.05 | Enolase (EC 4.2.1.11) (2-phosphoglycerate dehydratase) (2-phospho-D-glycerate hydro-lyase) (OSE1) | | |
| Gma.620.1.S1_at | CA820071 | Q2TUV6 | 1.68 | 0.05 | Glutathione S-transferase 2 | | |
| GmaAffx.93206.1.S1_x_at | CF808931 | Q2TUV6 | 2.01 | 0.03 | Glutathione S-transferase 2 | | |
| GmaAffx.83981.1.S1 at | CA937659 | Q9LFB4 | 1.95 | 0.03 | Hypothetical protein F7J8 200 | | |
| Gma.17388.1.S1_at | BG157586 | Q5ZBM2 | 2.20 | 0.03 | Hypothetical protein P0024G09.1 (Hypothetical protein P0019D06.13) | | |
| GmaAffx.70981.1.S1_at | BE801533 | Q3C1F4 | 1.77 | 0.05 | Nonsymbiotic hemoglobin | | |
| GmaAffx.31209.1.S1_at | BE822784 | Q9M1S8 | 1.77 | 0.04 | Probable glutamate carboxypeptidase 2 (EC 3.4.1721) (Probable glutamate carboxypeptidase II) | | |
| GmaAffx.26497.1.A1_at | BE657393 | Q9ZP18 | 2.39 | 0.03 | Purple acid phophatase (EC 3.1.3.2) (Fragment) | | |
| Gma.4280.1.S1 at | BQ297737 | Q6LA5 | 2.40 | 0.03 | Putative Sulfolipid synthase | | |
| GmaAffx.17098.1.S1_at | BM084976 | Q84ZV7 | 2.11 | 0.04 | R 12 protein | | |
| GmaAffx.6129.1.S1_at | BU547356 | Q6TF29 | 2.03 | 0.03 | Rapid alkalinisation factor 1 | | |
| GmaAffx.12369.1.A1_at | CD412820 | Q9FEG2 | 2.48 | 0.03 | TOM1 protein (Hypothetical protein At4g21790) | | |
| GmaAffx.6567.1.S1_at | CD403256 | Q9T081 | 3.70 | 0.01 | UDP rhamnose—anthocyanidin-3-glucosidde rhamosyltransferase-like protein | | |
